# Dynamics of cell-free tumor DNA correlate with early MRI response during chemoradiotherapy in rectal cancer

**DOI:** 10.1186/s13014-024-02540-4

**Published:** 2024-11-06

**Authors:** Kerstin Clasen, Cihan Gani, Leon Schuetz, Stephan Clasen, Nadja Ballin, Irina Bonzheim, Michael Orth, Stephan Ossowski, Olaf Riess, Maximilian Niyazi, Christopher Schroeder, Olga Kelemen

**Affiliations:** 1 Department of Radiation Oncology, University Hospital Tübingen, Tübingen, Germany; 2 Institute of Medical Genetics and Applied Genomics, University Hospital Tübingen, Tübingen, Germany; 3 Department of Diagnostic and Interventional Radiology, District Hospital Reutlingen, Reutlingen, Germany; 4 Institute of Pathology and Neuropathology, University Hospital Tübingen, Tübingen, Germany

**Keywords:** ctDNA, cfDNA, NGS, Biomarker, Adaptive radiotherapy, Imaging, Magnetic resonance imaging

## Abstract

**Background:**

In locally advanced rectal cancer, the prediction of tumor response during and after neoadjuvant treatment remains challenging. In terms of organ preservation, adaptive radiotherapy, and intensified (total) neoadjuvant therapies, biomarkers are desirable for patient stratification.

**Methods:**

In 16 patients, weekly blood samples (*n* = 86) to detect cell-free tumor DNA (ctDNA) during long-course neoadjuvant chemoradiotherapy were analyzed. Data were correlated with initial tumor volumes, MRI response in week 2 and 5 of radiotherapy as well as with pathologic tumor response after resection and outcome parameters.

**Results:**

Most patients showed decreasing ctDNA during the course of radiochemotherapy. However, we found heterogenous dynamics of ctDNA and could identify three groups: (1) decline (2) no clear decline and/or late shedding (3) persistence of ctDNA. In seven patients we could detect significant amounts of ctDNA in week 5 or week 6 of treatment. In our pilot cohort, we did not find significant correlations of ctDNA dynamics with pathologic response or outcome parameters. However, patients with distinct decline of ctDNA had larger tumor volumes prior to treatment, and MRI imaging in week 2 and 5 revealed bigger absolute decrease of tumor volumes. If significant levels of ctDNA were found in week 5 and / or 6, patients showed less absolute tumor volume decrease in week 2 and 5.

**Conclusions:**

Weekly measurement of ctDNA during radiochemotherapy is feasible and might represent a promising biomarker. Bigger initial primary tumors showed different ctDNA shedding profiles compared with smaller primary tumors and correlations of ctDNA dynamics with early imaging response were found.

**Supplementary Information:**

The online version contains supplementary material available at 10.1186/s13014-024-02540-4.

## Background

Since several years, a standard treatment option for patients with locally advanced rectal cancer (UICC stage II/III) implies neoadjuvant chemoradiotherapy (NCRT) and subsequent surgical resection followed by adjuvant chemotherapy [1]. Favourable 10-year overall survival rates of about 60% and local recurrence rates of 7% were reported [2]. Likewise, excellent locoregional results with combined modality treatment were reported by the German CAO/ARO/AIO-04 study, showing local control rates > 95% after 3 years [3]. However, to address distant failure rates and to support organ preservation strategies aiming for higher pathologic complete response (pCR) rates, intensified (total) neoadjuvant treatment regimes were recently published [4–6]. pCR defined as ypT0N0 of the resection specimen can be found frequently after neoadjuvant treatment [3, 7]. Therefore, approaches to achieve organ preservation by “watch and wait” strategies are upcoming and results are promising [6, 8, 9].

However, to support personalized therapy approaches, to predict tumor response and to estimate oncologic outcome, biomarkers are needed for patient stratification but have not been established in clinical routine so far. Yet, multi-omics and machine learning approaches might be useful to stratify patients in future and respective trials are ongoing [10].

Circulating cell-free tumor DNA (ctDNA) has been identified as a possible biomarker in colorectal cancer as these tumors shed relevant amounts of DNA into the blood [11, 12]. Thus, diverse applications of ctDNA as a biomarker have been suggested, such as early indication of tumor response in adjuvant or palliative treatments, molecular profiling and the detection of minimal residual disease (MRD) [11, 13, 14]. Moreover, in colon cancer, ctDNA-guided stratification for adjuvant treatment was successfully evaluated, recently [15].

In locally advanced rectal cancer, some reports about serial liquid biopsies have been published [16]. Samples were usually taken before and after neoadjuvant treatment [17–20] or rarely once [21–24] or twice (fraction 15 and 25 (last day) of NCRT) [25] during NCRT. There are hints, that serial sampling of ctDNA might be a promising biomarker to predict recurrence free survival in rectal cancer by detecting MRD in terms of distant metastases (i.e. minimal metastatic disease, MMD) [17]. However, the potential to predict pathologic response after NCRT (i.e. (minimal) residual local disease) has been discussed controversially [17].

Thus, our pilot biomarker study intended to investigate ctDNA dynamics during long-term NCRT by weekly sampling and to correlate these data with magnetic resonance imaging (MRI) during treatment as well as with pathologic response, and outcome parameters.

## Methods

Data was acquired prospectively and all patients declared their informed consent before participating in the study. The study was approved by the local ethics committee before recruitment started (734/2015BO2). Twenty patients with locally advanced rectal cancer (UICC stage II and III) were recruited for this pilot study. However, one patient was excluded before treatment start as therapy had to be modified due to an acute cardiac event. Three patients could not be evaluated for ctDNA dynamics owing to lacking tumor tissue in the formalin-fixed paraffin-embedded (FFPE) samples from pathology or insufficient ctDNA-detection in spite of deep sequencing. Thus, 16 patients could be evaluated in this pilot study. For pathologic response evaluation, the Dworak tumor regression grade (DW) of the resection specimen was recorded and grouped indicating bad (DW 1 + 2) versus good (DW 3 + 4) response. Clinical long-term follow-up was 56 months (median).

### Treatment schedule and imaging

For long-term neoadjuvant treatment, 50.4 Gy were delivered in 28 fractions by intensity-modulated radiotherapy (IMRT). For concurrent chemotherapy, two courses of 5-fluorouracil (5-FU) in treatment week 1 and 5 were applied. Six patient received additional treatment by deep regional hyperthermia (twice weekly, range: 2–10 sessions, mean: 8 sessions). Besides computed tomography (CT) to rule out metastatic disease, patients received standard magnetic resonance imaging (MRI) for local staging and treatment planning as well as additional MRI imaging (Magnetom Symphony, Siemens, Erlangen, Germany) of the pelvis during treatment (week 2 and week 5) to estimate tumor response. Tumor volumes were contoured on T2-weighted imaging by an experienced radiation oncologist (CG) and an experienced radiologist (SC).

### Blood sampling

**Fig. 1:**
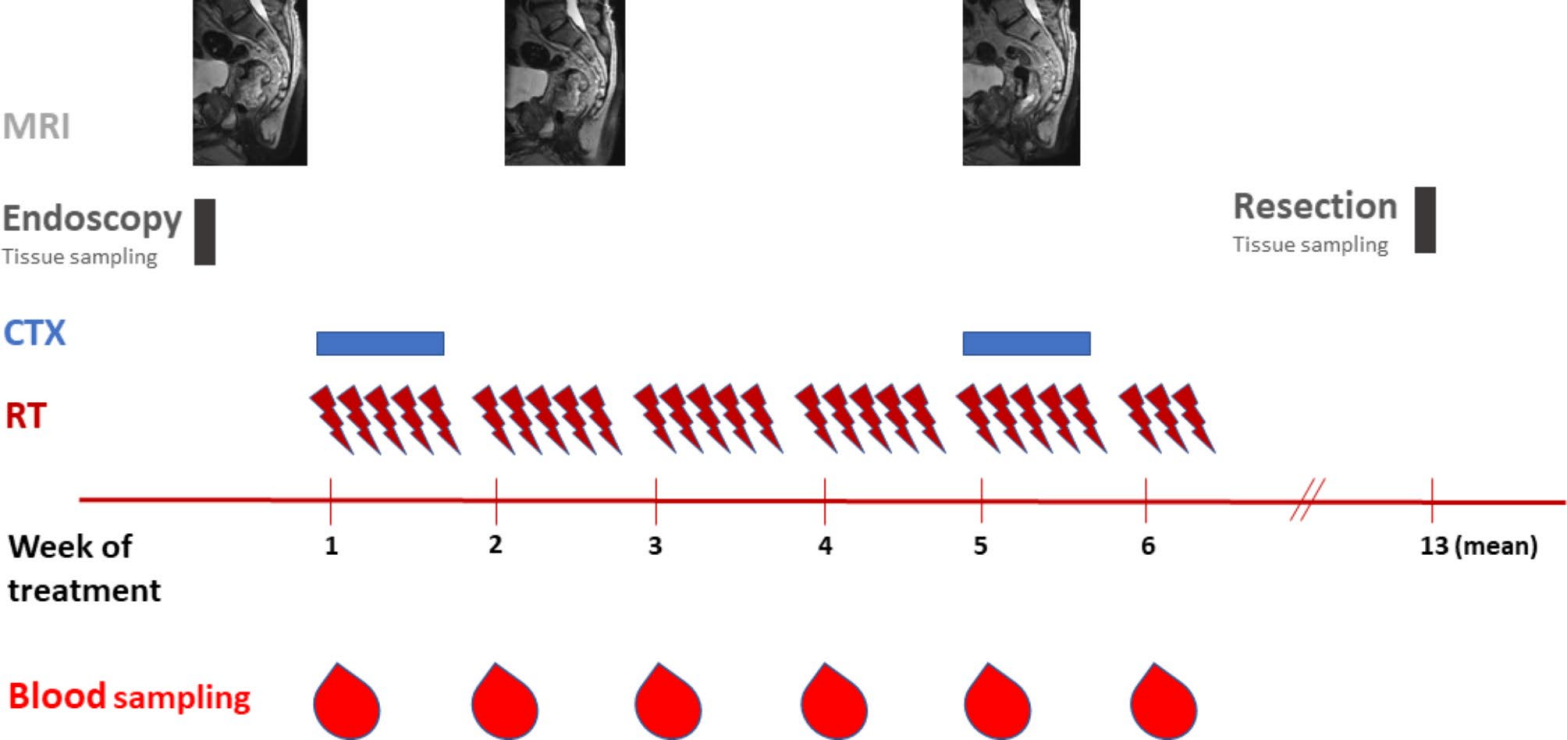
Study design. After diagnosis and endoscopy, all patients had pre-therapeutic magnetic resonance imaging (MRI). MRI imaging was repeated in week 2 and week 5. Radiotherapy (RT) and concomitant chemotherapy (CTX) were administered over 6 weeks. Blood samples for ctDNA monitoring were collected weekly (preferably on Mondays)

Blood samples were collected at the first day of treatment (pre-therapeutically as baseline sample) and weekly thereafter using EDTA tubes (Sarstedt, Nümbrecht, Germany). The samples were centrifuged twice and plasma was stored immediately at -80° Celsius. To rule out possible treatment-associated confounding factors, samples were usually collected on Mondays (after the weekend) before irradiation. The study design is visualized in Fig. 1.

### Tumor and ctDNA sequencing

Tumor and normal tissue were macro-dissected from one to ten 5 μm paraffin sections and DNA was extracted using the Maxwell RSC DNA FFPE Kit and the Maxwell RSC Instrument (Promega, Madison, WI, USA).

A Covaris E220 Ultrasonicatior was used to shear 200 ng genomic DNA into fragments of 150–200 bp (Covaris, Woburn, MA, USA). Fragmented DNA was end repaired, A-tailed, adaptor ligated, and amplified with Agilent’s SureSelect XT Low Input Target Enrichment System for Illumina Paired-End Multiplexed Sequencing Library kit following the manufacturer’s instructions (Agilent Technologies, Santa Clara, USA). A custom-designed hybrid capture panel covering 708 cancer related genes, selected promoter regions and fusions was used for target enrichment (Agilent Technologies, Santa Clara, USA). The libraries were sequenced on a NovaSeq6000 sequencing platform (Illumina, San Diego, CA, USA) in paired-end mode as specified by the manufacturer. The sequencing data was analyzed with the megSAP pipeline (https://github.com/imgag/megSAP).

To create tumor-informed target capture-panels, variant lists with up to 46 individual variants per patient were generated based on the tumor-normal sequencing results (supplemental file). The variants were filtered based on allele frequency, oncogenicity and considerations of sequencing quality. The selected variants and fingerprint SNVs for sample verification were used to design patient-specific oligo probes (NGS Discovery Pool, IDT, Coralville, USA).

The MagMAX™ Cell-Free DNA Isolation Kit (ThermoFischer Scientific, Waltham, USA) was utilized for cfDNA isolation. CfDNA quality was analyzed on the TapeStation with the Cell-free DNA ScreenTape (Agilent Technologies, Santa Clara, USA) and quantified using the Qubit dsDNA HS Assay Kit (ThermoFisher Scientific, Waltham, USA). NGS libraries were prepared from 2.9 to 111 ng of cfDNA using the xGenPrism DNA Library Prep Kit (IDT, Coralville, USA). For error correction and increased accuracy unique molecular identifiers (UMIs) were attached to the libraries prior to PCR amplification. Libraries were sequenced on a NovaSeq6000 (Illumina, San Diego, USA).

Sequencing data of cell-free DNA was analyzed with umiVar (https://github.com/imgag/umivar). For the alignment of reads we used bwa-mem (https://github.com/lh3/bwa) and reads were deduplicated using UMIs. Only reads with at least three duplicates were kept for further downstream analysis. The minimal residual disease was calculated using a Fisher exact test to compare the monitoring variants of each sample with the corresponding background noise in reference regions of the cell-free DNA.

### Data analysis

For analysis of ctDNA dynamics we choose two approaches: first the overall trend of ctDNA allele frequencies during NCRT and second the incidence of significant proof of ctDNA at the end of treatment (i.e. either in week 5 or week 6). Statistics were calculated using IBM SPSS Version 28. For correlations, the Mann-Whitney U-Test was used and outcome estimations were calculated by the Kaplan-Meier method and the log-rank test. *P*-values < 0.05 were considered statistically significant.

## Results

In our biomarker pilot study, we included two female and 14 male patients. Median age was 68 years at diagnosis (range 37–79 years). All patients had microsatellite-stable tumors and did not show hypermutation. Two patients achieved pCR after NCRT and five patients developed metastatic disease. No local relapse was recorded.

We could collect, process and analyze 86 blood samples. We recorded some drop outs (*n* = 10) at single timepoints due to clinical, logistic, technical or quality issues. All but one planned MR imaging could be conducted. Solely one MRI in week 5 was cancelled due to an acute pulmonary embolism.

**Fig. 2:**
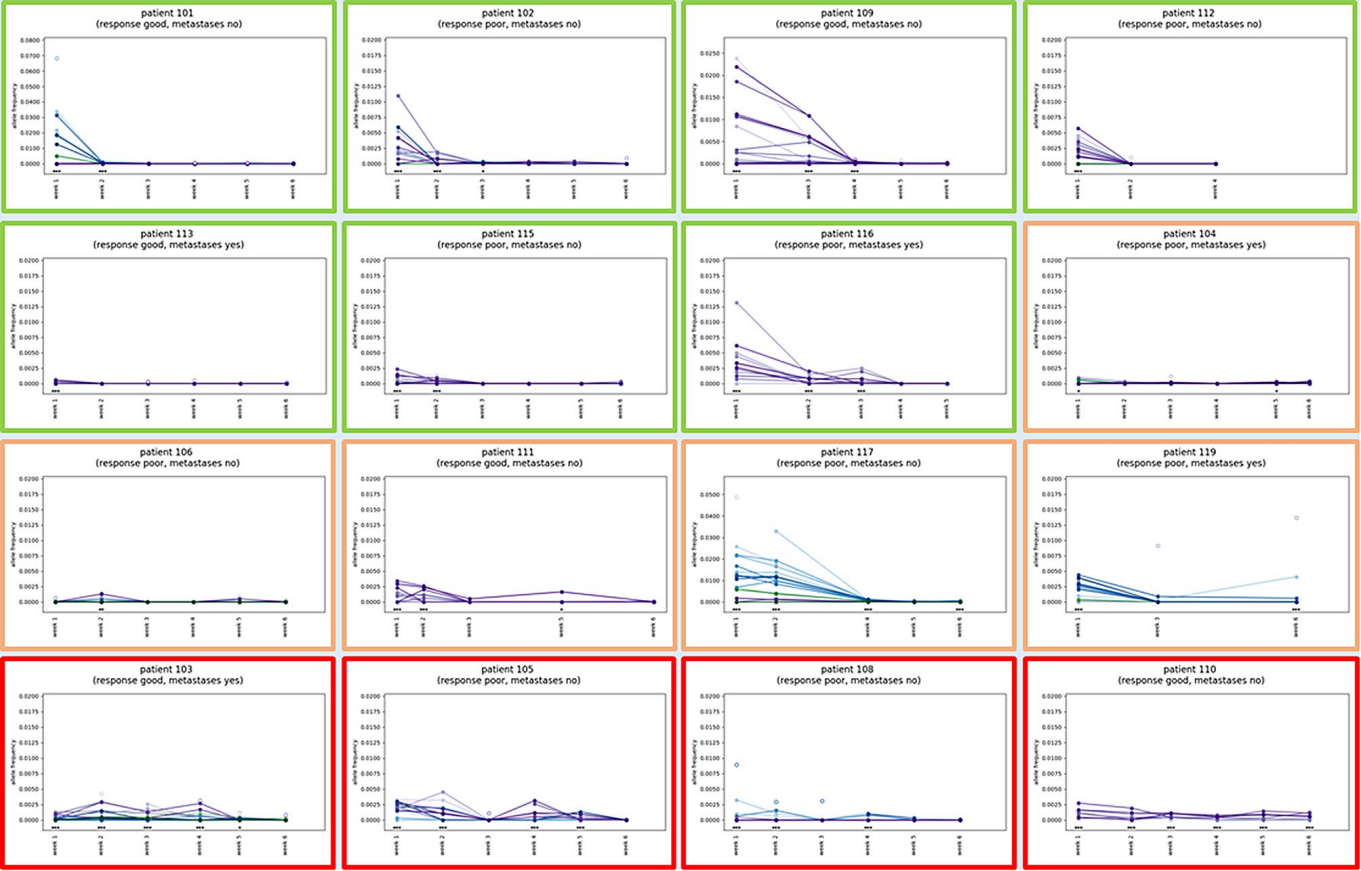
Dynamics of circulating cell-free tumor DNA (ctDNA) in 16 patients with associated patient features: good versus bad pathologic response (Dworak 1 + 2: bad; 3 + 4 good response) and the occurrence of metastases during follow-up. Patients are grouped accordingly to the respective ctDNA dynamics: green - decline; orange – no clear decline and/or late ctDNA shedding; red - persistence of ctDNA. Each line connects the respective allele frequencies of one particular variant that was tracked over time. Significant proofs of ctDNA (considering all monitored variants in the respective patient) are marked by asterisks (***: *p*-value < 0.001; **: *p*-value < 0.01; *: *p*-value < 0.05)

**Fig. 3:**
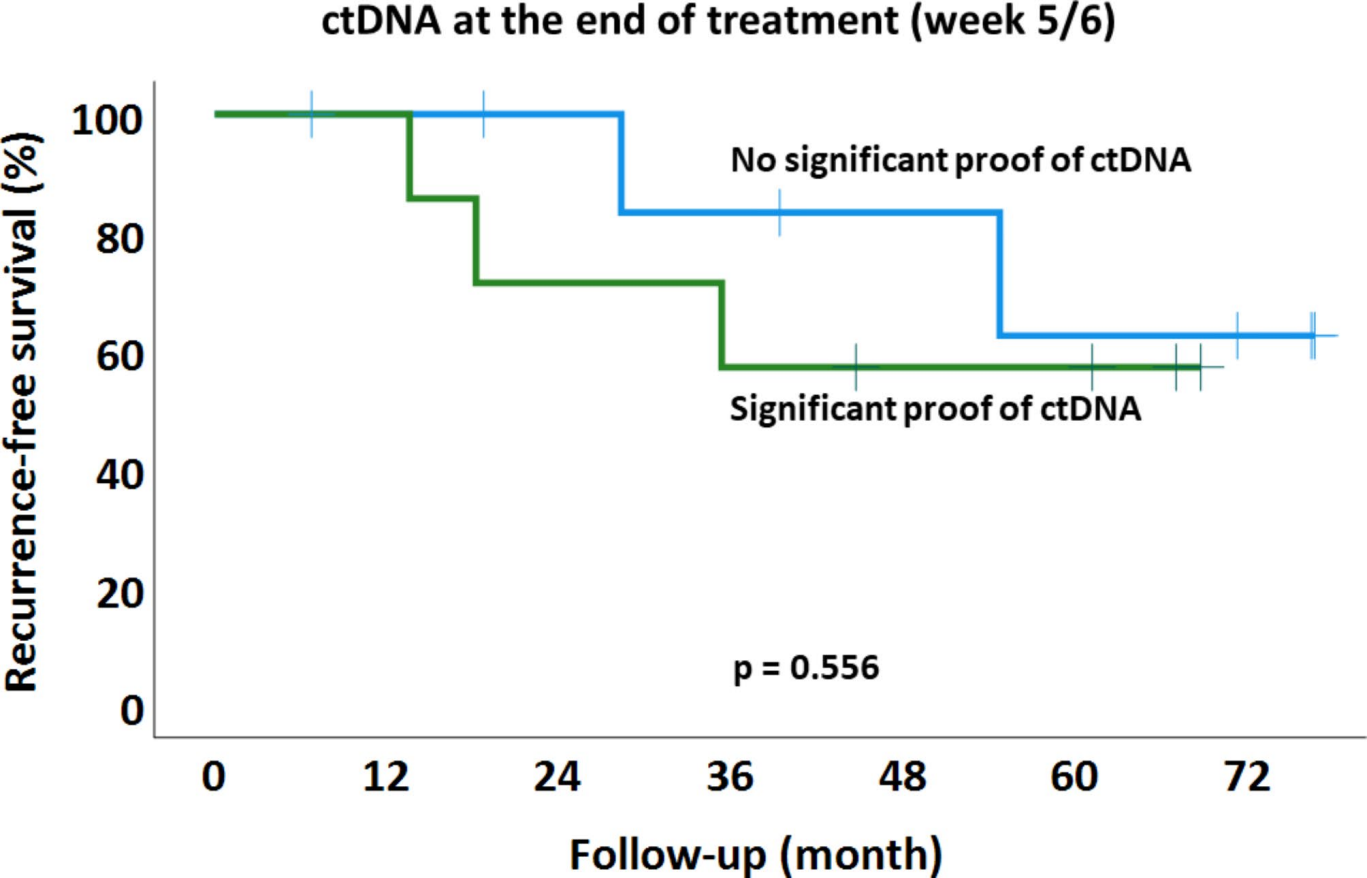
The proof of circulating cell-free tumor DNA (ctDNA) in the liquid biopsies at the end of treatment (week 5 or 6) was not significantly associated with tumor recurrence

ctDNA could be detected pre-therapeutically (week 1, baseline sample) in all patients except one (patient 106). ctDNA dynamics over time are visualized in Fig. 2. We found diverse patterns of ctDNA shedding and grouped these accordingly. First, we found patients with decline of ctDNA during NCRT. Second, some patients did not show clearly declining levels of ctDNA and/or had significant (asterisked* in Fig. 2) proof of ctDNA towards the end of treatment (“late shedding”). Third, we observed persistence of ctDNA in several patients. Two patients achieved pCR after NCRT. These patients showed either highly significant proof of ctDNA at all timepoints (patient 110, “persistence”) or significant proof of ctDNA in week 5 (patient 111; “late shedding”). We did not find correlations between pathologic response or long-term outcome parameters and ctDNA dynamics (data not shown). Significant or no significant ctDNA proof at the end of treatment (week 5 or 6) was also not associated with Dworak tumor regression grade (Table 1), recurrence-free survival (Fig. 3, *p* = 0.556) or overall survival (*p* = 0.529).

**Table 1:** Cross classification table for “proof of ctDNA at the end of neoadjuvant treatment (week 5 / 6)” and corresponding “Dworak tumor regression grades”. No clear correlation could be found. *P*-values are not provided due to small numbers

		Dworak regression grade				Total
		1	2	3	4	
Proof of ctDNA in week 5 / 6	No	2	3	3	0	8
	Yes	1	3	1	2	7
	Total	3	6	4	2	15

**Fig. 4:**
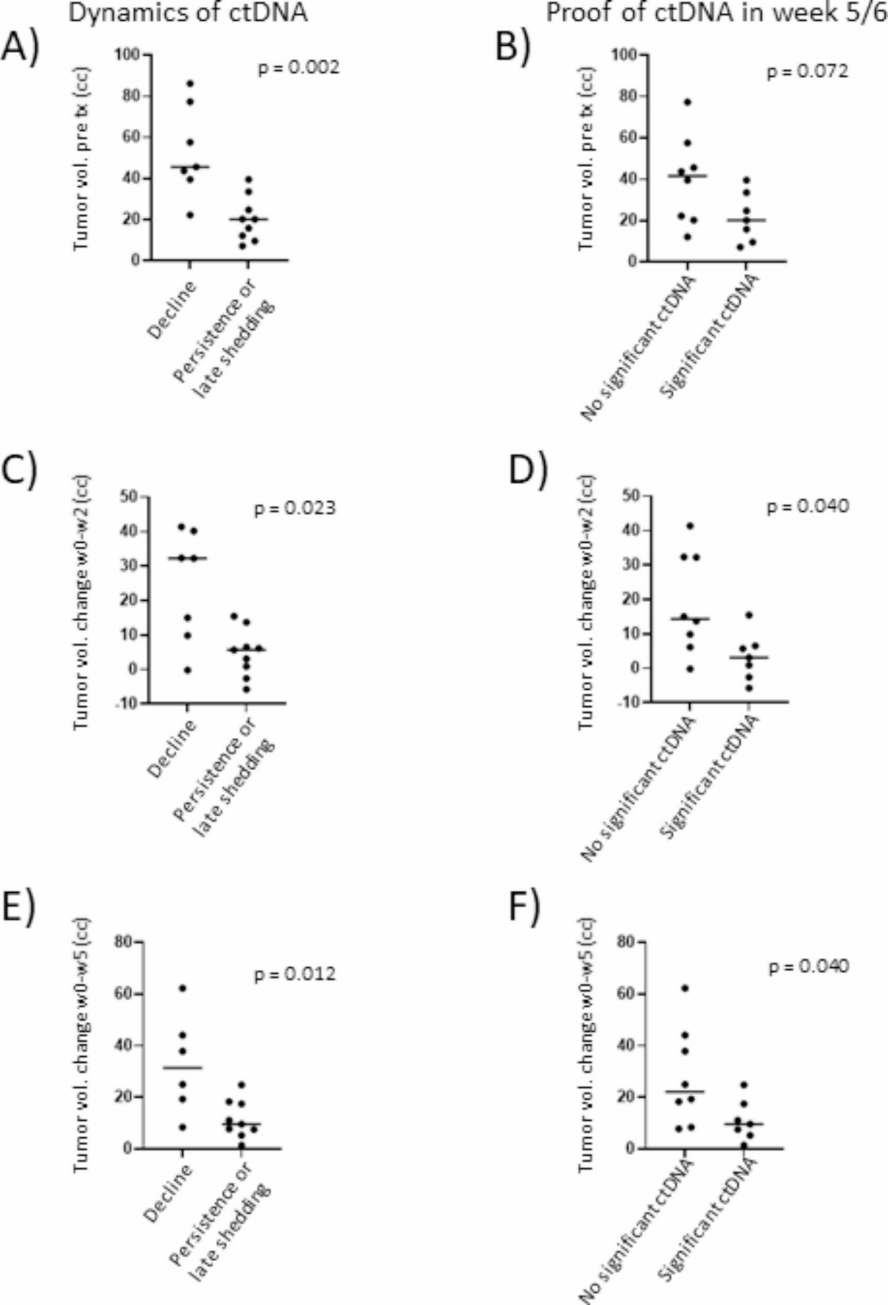
Correlations of dynamics of circulating cell-free tumor DNA (ctDNA) (“decline” versus “late shedding or persistence”) and the proof of ctDNA at the end of treatment in week 5 / 6 with the pre-therapeutic magnetic resonance imaging (MRI) volumes (tumor volumes pre tx (cc)) (**A** and **B**), the absolute tumor volume change in week 2 (**C** and **D**) and MRI tumor volume decrease between baseline and week 5 (**E** and **F**)

CtDNA dynamics and residual ctDNA in the liquid biopsies towards the end of NCRT (week 5 and/or 6) were correlated with the initial MRI tumor volumes and MR imaging-based tumor response in week 2 and week 5. Patients with declining courses of ctDNA presented with bigger pre-therapeutic tumor volumes (Fig. 4A, *p* = 0.002) whilst no difference of initial tumor volumes could be observed between patients with ctDNA persistence and patients with late ctDNA shedding (*p* = 0.975). Thus, these groups were merged. Furthermore, patients with declining ctDNA dynamics showed significantly higher absolute MRI tumor volume decrease (cc) in week 2 (Fig. 4C, *p* = 0.023) and week 5 (Fig. 4E, *p* = 0.012). Patients with proof of residual ctDNA in week 5 / 6 did not show significantly smaller initial tumor volumes (Fig. 4B, *p* = 0.072) but presented with impaired absolute tumor volume decrease in week 2 (Fig. 4D, *p* = 0.040) and week 5 (Fig. 4F, *p* = 0.040).

## Discussion

Our pilot biomarker study investigated ctDNA during NCRT for locally-advanced rectal cancer by weekly sampling. We correlated these dynamics with initial MR imaging and MRI response (in week 2 and 5 of NCRT) as well as with outcome parameters. In most previous studies, ctDNA samples were collected once or twice during neoadjuvant treatment or solely pre- and post NRCT [17–25]. Thus, our study provides valuable insights into ctDNA dynamics during treatment.

Using an ultra-deep sequencing tumor-informed approach, we had high baseline detection rates of ctDNA. By weekly monitoring we could observe diverse dynamics of ctDNA during the course of neoadjuvant treatment. In the majority of patients, ctDNA levels declined during NCRT and many patients showed clearance of ctDNA after week 3 or 4. However, in some patients, after initial ctDNA decline we observed “late shedding” of ctDNA in the last weeks of treatment or even persistence of ctDNA throughout NCRT. Thus, we found various patterns of ctDNA shedding during radiochemotherapy which might reflect variable biological treatment responses.

In line with our results, a rapid decrease of ctDNA after onset of NCRT was reported in previous studies that investigated samples during neoadjuvant treatment (Zhou et al.: baseline: 75% proof of ctDNA; 2–3 weeks after NCRT initiation: 15.6% [21]; Khakoo et al.: baseline: 74% proof of ctDNA; mid NCRT: 21% [22]). This highlights the effectiveness of NCRT in rectal cancer and the potential of ctDNA as a concomitant biomarker. In future studies we suggest frequent sampling especially during the first weeks and towards the end of NCRT for further elucidation of ctDNA dynamics.

Our biomarker-study did not reveal significant correlations between dynamics of ctDNA during NCRT and pathologic response or long-term outcome of our patients. However, the small cohort size has to be considered as a limiting factor. To date, the potential of ctDNA monitoring to predict pathologic response after NCRT is still under debate and the majority of previous reports failed to proof associations [16] whilst the potential to monitor MRD after the end of treatment seems promising. A recent study by Vidal et al. evaluated ctDNA samples before and after total neoadjuvant treatment (tumor-agnostic assay) [17]. A correlation of ctDNA with pCR or ypT or ypN status could not be found. However, if ctDNA could be measured in the pre-surgery sample, a higher rate of distant recurrence and impaired overall survival was observed during follow-up [17]. Tie et al. collected liquid biopsies at baseline, 4–6 weeks after NCRT and post-surgery in 159 patients and assessed one variant per patient over time [18]. No significant association with pCR rates could be found but patients with ctDNA proof after NCRT or post-surgery had dismal recurrence-free survival. The study of Khakoo et al. included 47 patients and investigated ctDNA pre-, mid- (week 3 or 4) and post-NCRT (4–12 weeks after NCRT) as well as post surgery by monitoring up to three variants per patient [22]. An association of persistent ctDNA and the occurrence of metastases was reported. Three patients achieved pCR. In these patients, ctDNA was only detectable pre-NCRT. Apart from that observation, no significant correlations of ctDNA during NCRT or pre-surgery with pathologic response were found. In contrast, two Chinese studies report correlations of ctDNA clearance and pathologic response (pCR) [23, 25].

Challenges to compare studies and respective results imply the various approaches to detect ctDNA regarding timepoints of sampling, tumor-informed versus tumor-agnostic assays, the number of tracked variants and possible detection limits as well as heterogenous cohorts and diverse treatment regimes. Thus, the potential of ctDNA to predict pathologic response is still under investigation and especially in upcoming organ preservation strategies, further exploration of biomarkers like ctDNA with ultra-sensitive approaches is desirable. ctDNA as a biomarker for oncologic long-term outcome appears promising especially in samples after completion of treatment to monitor MRD.

Besides pathologic response in the resection specimen, we correlated the courses of ctDNA with baseline MRI and imaging response during treatment in week 2 and week 5. In this way, we investigated ctDNA as a marker to monitor treatment-response whilst neoadjuvant therapy was ongoing. Interestingly, we found a higher number of ctDNA decline over time in larger primary tumors. Furthermore, the absolute image-based tumor regression (cc) between baseline and week 2 as well as baseline and week 5 was positively correlated with ctDNA clearance during NCRT. Underlying mechanisms are unclear to date and further investigations are needed. To date, data relating ctDNA to MRI features in NCRT for rectal cancer are sparse.

Khakoo et al. correlated liquid biopsies (pre-, mid-, post-NCRT, post-surgery) with MRI response (3–6 weeks after completion of NCRT) [22]. By RECIST measurement, no association with ctDNA detection rate was seen at any time. However, the MRI tumor regression grade (mrTRG) revealed detectable ctDNA after completion of NCRT to be associated with poor mrTRG response whilst other timepoints did not correlate with mrTRG.

In a further report, the benefit of incorporating both, ctDNA features and mrTRG as complementary tools to predict pCR was suggested [25].

Zhou et al. investigated ctDNA at four times: pre- and during-NCRT as well as pre- and post-surgery [21]. Baseline detection of ctDNA was associated with baseline MRI extramural vascular invasion (EMVI) status. No correlations of ctDNA measurements pre-NCRT or 2–3 weeks after onset of NCRT and MRI response (“postneoadjuvant MRI”) were found. However, a correlation between the pre-surgical ctDNA evaluation and post-neoadjuvant MRI-defined EMVI score was reported.

Thus, the combined investigation of ctDNA and MRI features seems promising for further personalized approaches in the management of locally advanced rectal cancer.

The strength of our study is the prospective character, the mainly homogenous treatment, long period of follow-up and the tumor-informed assay based on initial tumor tissue sequencing analyzing 708 oncogenes. Therefore, in each patient multiple variants could be monitored. In contrast to others, we did not only consider the variant with the highest initial allele frequency (at baseline) [18, 25], or 1–3 variants [22] for ctDNA monitoring over time, but included all variant positions in a statistical test to determine significant residual disease. Furthermore, weekly monitoring enabled a detailed view on ctDNA dynamics and correlations with corresponding MR imaging during treatment. Weakness of our study is the relatively small cohort and a potential confounder by treatment of some patients with additional deep regional hyperthermia wherefore our results are hypothesis-generating but need to be confirmed in larger studies. Furthermore, as ultra-deep sequencing approaches are needed to detect very low tumor burden we cannot rule out detection limits with our current method despite of sequencing with a raw depth of up to 35,000x. We reported potential confounding factors like acute infections or application of Granulocyte Colony-Stimulating Factor (G-CSF) for the interpretation of ctDNA dynamics before [26]. In the recent cohort, our patients did not suffer from relevant infections or toxicities during sampling (e.g. we did not collect blood samples of the patient 112 with acute pulmonary embolism after this event any more). However, yet unknown confounders during NCRT cannot be ruled out.

## Conclusions

In our weekly sampling approach, we found divergent dynamics of ctDNA shedding during NCRT for locally advanced rectal cancer. Furthermore, MRI tumor size and absolute tumor regression (cc) at two times during treatment could be correlated with patterns of ctDNA decline and clearance. These findings are hypothesis-generating and might reflect diverse biological tumor features impacting treatment response and ctDNA shedding during chemoradiation which need to be elucidated in future studies. If correlations can be confirmed, complementary ctDNA and MRI data might help to further develop biomarker-driven personalised-medicine approaches in high-risk patients or organ preservation studies.

## Electronic supplementary material

Below is the link to the electronic supplementary material.

Supplementary Material 1

## Data Availability

The datasets used and analyzed during the current study are available from the corresponding author on reasonable request.
